# EFFICACY AND TOLERABILITY OF CEFDITOREN PIVOXIL IN UNCOMPLICATED SKIN AND SKIN STRUCTURE INFECTIONS IN INDIAN PATIENTS

**DOI:** 10.4103/0019-5154.57612

**Published:** 2009

**Authors:** Charu Manaktala, Amit Kumar Singh, Manish Verma, Asheesh Sachdeva, Himanshu Sharma, Arjun Roy, R K Jalali, R Gowrishankar, A Kumar, A Sainath Kumar, A M Jayaraman, B Swarnkar, C R Srinivas, Chitra Nayak, D Duttaroy, D Umrigar, Madhuri Jesudanam, N Maheshwari, P Shetty, R P Singh, S Ghate, S Sacchidanand, S Tolat, Salman Bhoira, Y Marfatia

**Affiliations:** 1 *Medical Affairs and Clinical Research, Ranbaxy Laboratories Ltd, 77-B, IFFCO Road, Sector-18, Udyog Vihar Industrial Area, Gurgaon, Haryana*; 2 *Department of Dermatology, Kamineni Institute of Medical Sciences, Naketpally, Nalgonda*; 3 *Princess Esra Hospital, Hyderabad*; 4 *Department of Dermatology, Stanley Govt Hospital, Chennai*; 5 *Anand Bazar Main Road, Indore*; 6 *Department of Dermatology, PSG Hospitals, Coimbatore*; 7 *BYL Nair Charitable Hospital & TNMC, Mumbai*; 8 *Department of Surgery, Govt. Medical College & Shri Sayajirao General Hospital, Vadodara*; 9 *Department of Dermatology and STD, GMC and New Civil Hospital, Surat*; 10 *Flora Apartments, Besides Almond House, Road No. 3, Banjara Hills, Hyderabad*; 11 *Hari Clinic, 3/95, Kamal Kunj, Vikas Nagar, Lucknow*; 12 *Raghu Skin Clinic, Suvarna Complex, Karver Road, Kothrud, Pune*; 13 *Department of Dermatology, Govt. Medical College, Nagpur*; 14 *Foundation for Medical Research, Near Flora Dinning Restaurant, Worli Sea Face, Mumbai*; 15 *Department of Skin & STD, Victoria Hospital, Bangalore Medical College, Bangalore*; 16 *Skin & VD, Shangrila Garden, Bund Garden Corner, Pune*; 17 *Dr. Shaukat's Nursing Home, Natawala Building, Cadell Road, Mahim (W), Mumbai*; 18 *Department. of Skin & VD, Medical College & SSG Hospital, Vadodara.*

**Keywords:** *Cefditoren pivoxil*, *uncomplicated skin and skin structure infection*, *uSSSI*

## Abstract

**Background:**

Uncomplicated skin and skin structure infections (uSSSI) are commonly encountered community-acquired infections and are typically confined to the superficial layers of the skin. Hence, they seldom lead to the destruction of skin structures.

**Aims:**

To evaluate the efficacy and tolerability of cefditoren pivoxil in uSSSI in Indian patients.

**Methods:**

One hundred and seventy-eight patients diagnosed with uncomplicated SSSI were enrolled in this randomized, comparative, multicentric study. Patients received either cefditoren pivoxil or cefdinir for ten days. Efficacy was assessed both clinically and microbiologically. Safety evaluation consisted of reporting of type, frequency, severity, and causal relationship of adverse events.

**Results:**

One hundred and fifty-one patients completed the study. Clinical and bacteriological efficacy of cefditoren pivoxil was comparable to that of cefdinir in the treatment of uSSSI. One hundred and five patients were eligible for per protocol (PP) analysis of bacteriological outcome and clinical efficacy. Clinical cure or improvement was achieved in 98.00% patients treated with cefditoren pivoxil and 98.18% patients treated with cefdinir. In the modified Intent to Treat (mITT) patient population, clinical cure or improvement was recorded in 97.33% patients treated with cefditoren pivoxil and 96.20% patients treated with cefdinir. Microbiological eradication (or presumed eradication) was recorded in 88.00% patients treated with cefditoren pivoxil and 94.55% patients treated with cefdinir. The above differences in the outcome rates between the two drugs were not statistically significant. Six adverse events (AEs) (two in cefditoren group and four in cefdinir group) were reported in this study.

**Conclusion:**

Cefditoren pivoxil 200 mg b.i.d. was effective and well tolerated in the treatment of uSSSI.

## Introduction

Uncomplicated skin and skin structure infections (uSSSI) are commonly encountered community-acquired infections. The majority of uSSSIs are caused by aerobic Gram-positive cocci, specifically *Staphylococci* and *Streptococci*.[1] As these infections are typically confined to the superficial layers and seldom lead to the destruction of skin structures and resultant systemic dissemination, they can be treated with an oral antibiotic with potent microbiologic activity against Gram-positive pathogens. Cefditoren pivoxil is a third-generation, oral cephalosporin with a broad spectrum of activity covering pathogens that are commonly implicated in uSSSI.[2] It is active against both Gram-positive and Gram-negative bacteria and is stable to hydrolysis by many common β-lactamases (*e.g*., TEM-1, ROB-1, SHV-1, SHV-3, SHV-10, OXA-5, OXA-12, PSE-1, PSE-2, PSE-3, PSE-4, SAR-1, HMS-1, CARB-4, LCR--1, TLE-1, and OHIO-1). In addition to the cephem nucleus common to all cephalosporins, cefditoren possesses a methylthiazolyl group that makes the drug active against Gram-positive organisms.[3] *In vitro* studies conducted in Europe, Japan, and USA have demonstrated that cefditoren is active against clinically relevant Gram-positive and Gram-negative organisms. MIC_90_ values for cefditoren against methicillin-sensitive. *aureus* ranged from 0.5 to 1 mg/L, and were similar to those seen with cefuroxime and cefdinir. However, MIC_90_ values for cefditoren were lower than those for cefpodoxime (2 to >4 mg/L), cefaclor (4-8 mg/L), and cefixime (>4-8 mg/L). The oral bioavailability of cefditoren pivoxil is 50-70% in the fed state, and the drug is well tolerated; the most common side effects observed in clinical trials were diarrhea and nausea.[3] The purpose of the present study was to compare the clinical cure rates, bacteriological eradication rates, safety, and tolerability of cefditoren pivoxil (200 mg *b.i.d.*) *vs*. cefdinir (300 mg *b.i.d*.) in Indian patients with uSSSI.

## Materials and Methods

This was a randomized, comparative, open-label, multicentric study in which 178 patients were enrolled across 17 centers in India. These patients were randomized to ten days of treatment with cefditoren 200 mg tablets (test drug) or cefdinir 300 mg capsules (reference drug) (manufactured by Ranbaxy Laboratories Ltd. India) b.i.d. Randomization was stratified centerwise and the schedule was prepared by using the PROC PLAN of SAS® system version 8.2 for windows (SAS Institute Inc., Cary, NC, U.S.A.). The protocol was approved by the Ethics committees of the Institutions. The study was performed in accordance with the Declaration of Helsinki and was consistent with the principles of Good Clinical Practice. Written informed consent was obtained from all the participants.

### Inclusion criteria

Patients included in the study were outpatients of either sex, ≥18 years of age, with acute onset (< 3 weeks duration) uncomplicated skin, skin structure, or soft tissue infection evidenced by erythema, swelling, pain, drainage, or other clinical signs. The uncomplicated skin, skin structure or soft tissue infection constituted any of the following: simple abscesses, impetiginous lesions, furunculosis, cellulitis, erysipelas, folliculitis, paronychia, traumatic, or postsurgical superficial wound infection. All patients were to have a microbiological specimen (infected material) obtained from the skin lesion(s) prior to the initiation of therapy.

### Exclusion criteria

Patients with a history of hypersensitivity to cefditoren pivoxil, cefdinir, penicillin, or the cephalosporin group of antibiotics; chronic/underlying skin condition at the site of infection involving prosthetic material; secondarily infected atopic dermatitis, eczema, thermal injury or acne vulgaris; solitary furuncle; skin and soft tissue infection with suspected or proven contiguous bone, nail bed and scalp involvement; requiring treatment with other systemic antibacterial drugs; clinically significant renal, hepatic, cardiac, hematological, gastrointestinal, or neurological disorder; abnormal laboratory values at the time of admission into the study: serum creatinine > 1.2 mg/dL, SGOT or SGPT > 1.5 times the upper limit of normal values, alkaline phosphatase or serum bilirubin > 1.2 times the upper limit of normal; and unstable concomitant disease or underlying conditions compromising the ability to respond to a bacterial infection, were excluded from the study.

Pregnant or breast-feeding women or women in the reproductive age group and not using reliable contraception, *i.e*., a medically accepted effective method of birth control; patients who had received antimicrobial therapy in the last 72 hours prior to enrollment; patients receiving antacids, iron supplements, H2-receptor antagonists and probenecid; patients who had participated in any other investigational study in the last one month; patients unwilling to give informed consent or unable to comply with study procedures; and patients who had been previously enrolled in the study, were also excluded from the study.

Patients made three clinic visits during the trial: on entry into the study (Day 0 or Visit 1), after three days of treatment (Day 3 or Visit 2), and after completion of therapy (Day 10-12 or Visit 3). Apart from assessments, specific signs and symptoms evaluated included fever, chills, malaise, pruritus, pain at the site of lesion(s), erythema around the lesion(s), induration, tenderness, regional lymphadenopathy, number of lesions, diameter of the largest lesion, ulceration of lesion(s), discharge from lesion(s), and crust/scab formation over the lesion(s). Material was obtained for bacterial culture from the skin lesion(s) by either needle aspiration or with a sterile swab. Swab specimens were obtained directly from the draining lesion in order to minimize contamination with uninvolved skin flora. Interventions such as incision and drainage of an abscess, daily debridements, or dressing changes were allowed at the discretion of the investigator. However, no local antimicrobials were allowed.

Specific signs and symptoms were evaluated on Visit 2 (Day 3) and on Visit 3 (Day 10-12) in a similar manner as at Visit 1. In addition, healing of lesions and appearance of new lesions, if any, were recorded at Visits 2 and 3. Microbiological evaluation was repeated at the end of study, if an appropriate site to culture was available.

The primary efficacy measure was clinical outcome that was based on clinical assessments at the end-of-treatment visit in the population that was clinically evaluable for efficacy. Two types of population were defined for clinical evaluation of efficacy: Per Protocol (PP) and modified Intent to Treat (mITT).

Per Protocol clinically evaluable patient population: These were patients who met inclusion/exclusion criteria; did not receive concomitant antimicrobial therapy; had documented evidence of infection with a bacterial pathogen, susceptible to study medication at baseline; and who complied with the dosing regimen or at least received three full days of therapy for patients deemed to be failing while on therapy. In patients who were withdrawn from the study as therapeutic failures, data from the clinical and microbiological assessments that were performed at the time of withdrawal were carried forward to the end-of-treatment visit.

Modified Intent to Treat clinically evaluable patient population: These were patients included in the per-protocol analysis and those who were culture-negative or had pathogen(s) resistant to study medication at baseline.

The clinical outcome was classified as follows:

### Clinical cure

Total resolution of all signs and symptoms of the infection and associated with complete healing of lesions (*i.e*., lesions disappear or are completely dry).

### Clinical improvement

Resolution of most of the signs and symptoms of infection associated with incomplete healing of lesions (*i.e*., lesions are either less extensive or only some lesions have dried) and no further antimicrobial therapy is necessary.

### Clinical failure

No improvement of clinical symptoms or lesions.

The secondary efficacy measure was microbiological outcome. To be considered microbiologically evaluable, patients had to be clinically evaluable (as described above) and show growth of pathogens on an adequate pre-treatment culture; repeat culture and antimicrobial susceptibility testing were done (if an appropriate site to culture was available) and the pathogen(s) was susceptible to the study medication at baseline.

Microbiological outcome was classified as follows:

### Eradication

No growth of the pre-treatment pathogen on a post-therapy culture.

### Presumed eradication

A post-therapy culture was not obtained due to lack of culturable material, secondary to an adequate clinical response.

### Failure

Lack of eradication of the initial pathogen for a subsequent culture at the end of treatment.

### Super infection

Eradication of the initial pathogen plus the isolation of one or more new pathogens at the end of treatment.

Safety evaluation was done for all the patients recruited into the study and consisted of reporting of type, frequency, severity, and causal relationship of adverse events.

### Analyses of data

Statistical analysis was performed using Fisher's Exact test for clinical and microbiological outcomes. In addition, 95% Confidence Interval (CI) of the mean difference of Test drug – Reference drug (T – R) was calculated. The proportion of patients with clinical cure or clinical improvement and the proportion of patients with bacteriological eradication or presumed eradication and adverse events have been reported. Clinical outcome has been reported for both Per Protocol (PP) and Modified Intention-To-Treat (mITT) patient populations.

## Results

### Disposition of patients

Of the 178 patients enrolled, 125 (70.22%) were male and 53 (29.78%) were female. Their mean age was 34.43 ± 13.49 years (range: 16 to 80 years). The demographic characteristics of the patients enrolled are presented in Table 1. All patients enrolled in this study were issued study medication (either cefditoren pivoxil or cefdinir) and were evaluable for safety. Of the 178 patients enrolled, 151 patients who completed the study and three patients who were withdrawn at Visit 2 for lack of clinical efficacy, were included in the mITT analysis of clinical outcome. Of the mITT population, 105 patients were eligible for PP analysis of clinical and bacteriological outcomes.

**Table 1:** Demographic characteristics of patients enrolled (n = 178)

Parameter	Cefditoren pivoxil (90)	Cefdinir (88)	*P* value
Gender (no. and %)			
Male	66 (73.33)	59 (67.05)	0.4136
Female	24 (26.67)	29 (32.95)	
Age (yrs)			
Mean ± SD	33.92 ± 14.85	34.94 ± 12.01	0.6143
Range	(17.00-80.00)	(16.00-73.00)	
Height (cm)			
Mean ± SD	160.98 ± 7.94	159.77 ± 8.56	0.3200
Range	(138.00-178.00)	(140.00-186.00)	
Weight (kg)			
Mean ± SD	61.80 ± 10.42	59.19 ± 9.49	0.0817
Range	(42.00-98.00)	(42.00-83.40)	

### Diagnoses and lesion characteristics at study entry

The demographic characteristics, diagnoses and the lesion characteristics at study entry were comparable across the two treatment groups. The majority of the infections were spontaneous [Table 2]. Furunculosis, simple abscess and folliculitis were the most common diagnoses in the patients enrolled in this study [Table 3]. All patients had superficial involvement of skin and skin structures.

**Table 2:** Cause of infection (n = 178)

Cause of infection*	Cefditoren pivoxil (90) (%)	Cefdinir (88) (%)	Total
Spontaneous	76 (84.44)	71 (80.68)	147
Trauma	10 (11.11)	13 (14.77)	23
Post-surgical	2 (2.22)	2 (2.27)	4
Bite	0 (0)	3 (3.41)	3

* Data NA for 2 patients in cefditoren pivoxil group, 2 causes marked for 1 patient in cefdinir group

**Table 3:** Diagnosis at admission (n = 178)

Diagnosis*	Cefditoren pivoxil (90) (%)	Cefdinir (88) (%)	Total
Simple abscess	19 (21.11)	22 (25.00)	41
Impetiginous lesion	11 (12.22)	8 (9.09)	19
Furunculosis	27 (30.00)	29 (32.95)	56
Folliculitis	15 (16.67)	17 (19.32)	32
Superficial post surgical wound infection	1 (1.11)	1 (1.14)	2
Cellulitis	6 (6.67)	4 (4.55)	10
Erysipelas	1 (1.11)	1 (1.14)	2
Paronychia	2 (2.22)	2 (2.27)	4
Superficial traumatic wound infection	8 (8.89)	6 (6.82)	14
Other	1 (1.11)	3 (3.41)	4

* 6 patients had more than 1 diagnosis

### Signs and symptoms at study entry in patients evaluable for efficacy

Signs and symptoms at study entry are presented in Tables 4 and 5. Pain at the site of lesion(s), erythema, tenderness and discharge from lesions were the most frequent signs and symptoms.

**Table 4:** Signs and symptoms on study admission in mITT population (n = 154)*

Signs and symptoms	Cefditoren pivoxil (75) (%)	Cefdinir (79) (%)
Fever		
Present	16 (21.33)	13 (16.46%)
Absent	59 (78.67)	66 (83.54)
Chills		
Present	0 (0)	2 (2.53)
Absent	75 (100)	77 (97.47)
Malaise		
Present	14 (18.67)	9 (11.39)
Absent	61 (81.33)	70 (88.61)
Pruritus		
Present	16 (21.33)	25 (31.65)
Absent	59 (78.67)	54 (68.35)
Pain at the site of lesion(s)		
Present	69 (92.00)	72 (91.14)
Absent	6 (8.00)	7 (8.86)
Erythema around the lesion(s)		
Present	66 (88.00)	71 (89.87)
Absent	9 (12.00)	8 (10.13)
Tenderness		
Present	70 (93.33)	72 (91.14)
Absent	5 (6.67)	7 (8.86)
Regional lymphadenopathy		
Present	18 (24.00)	17 (21.52)
Absent	57 (76.00)	62 (78.48)
Ulceration of lesion(s)		
Present	28 (37.33)	22 (27.85)
Absent	47 (62.67)	57 (72.15)
Discharge from lesion(s)		
Present	57 (76.00)	59 (74.68)
Absent	18 (24.00)	20 (25.32)
Crust/Scab formation		
Present	30 (40.00)	27 (34.18)
Absent	45 (60.00)	52 (65.82)
Induration		
Present	38 (50.67)	33 (41.77)
Absent	37 (49.33)	46 (58.23)

* Data presented as no. of patients with % of patients indicated within the brackets

**Table 5:** Signs and symptoms on study admission in the PP population (n = 105)*

Signs and symptoms	Cefditoren pivoxil (50) (%)	Cefdinir (55) (%)
Fever		
Present	10 (20.00)	9 (16.36)
Absent	40 (80.00)	46 (83.64)
Chills		
Present	0 (0)	1 (1.82)
Absent	50 (100)	54 (98.18)
Malaise		
Present	6 (12.00)	3 (5.45)
Absent	44 (88.00)	52 (94.55)
Pruritus		
Present	11 (22.00)	18 (32.73)
Absent	39 (78.00)	37 (67.27)
Pain at the site of lesion(s)		
Present	45 (90.00)	51 (92.73)
Absent	5 (10.00)	4 (7.27)
Erythema around the lesion(s)		
Present	44 (88.00)	50 (90.91)
Absent	6 (12.00)	5 (9.09)
Tenderness		
Present	48 (96.00)	51 (92.73)
Absent	2 (4.00)	4 (7.27)
Regional lymphadenopathy		
Present	10 (20.00)	12 (21.82)
Absent	40 (80.00)	43 (78.18)
Ulceration of lesion(s)		
Present	19 (38.00)	16 (29.09)
Absent	31 (62.00)	39 (70.91)
Discharge from lesion(s)		
Present	39 (78.00)	40 (72.73)
Absent	11 (22.00)	15 (27.27)
Crust/Scab formation		
Present	22 (44.00)	19 (34.55)
Absent	28 (56.00)	36 (65.45)
Induration		
Present	21 (42.00)	23 (41.82)
Absent	29 (58.00)	32 (58.18)

* Data presented as no. of patients with % of patients indicated within the brackets

### Bacteria isolated at study admission

All but two of the 178 patients had their lesion exudates cultured at study entry. Culture was negative in 18 patients (10.11%). *Staphylococci* were the most commonly isolated pathogens [Table 6].

**Table 6:** Bacteria isolated from skin lesions at study entry (n = 178)*

Pathogens	Cefditoren pivoxil (n = 90) (%)	Cefdinir (n = 88) (%)
*Staphylococcus aureus* or other spp.	73 (81.1)	67 (76.14)
*Streptococcus* spp.	5 (5.56)	4 (4.55)
*Klebsiella* spp.	1 (1.11)	2 (2.27)
*E. coli*	5 (5.56)	5 (5.68)
*Pseudomonas* spp.	3 (3.33)	0 (0)
*Corynebacterium* spp.	1 (1.11)	0 (0)
*Proteus* spp.	2 (2.22)	1 (1.14)
*Acinetobacter* spp.	1 (1.11)	1 (1.14)
*Enterobacter* spp.	1 (1.11)	0 (0)
Gram positive bacilli	0 (0)	1 (1.14)
Gram negative bacilli	0 (0)	2 (2.27)
Culture negative	6 (6.67)	12 (13.64)

* Data presented as no. of patients with % of patients indicated within the brackets; 2 organisms were isolated in 17 patients; 2 patients did not have culture at study admission

#### Efficacy

**Figure 1:**
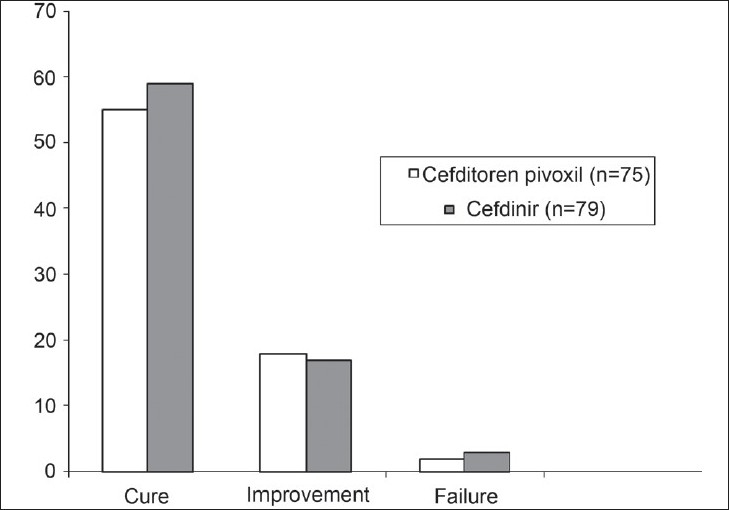
Clinical efficacy of cefditoren pivoxil and cefdinir in the modified intent to treat population (n = 154)

**Figure 2:**
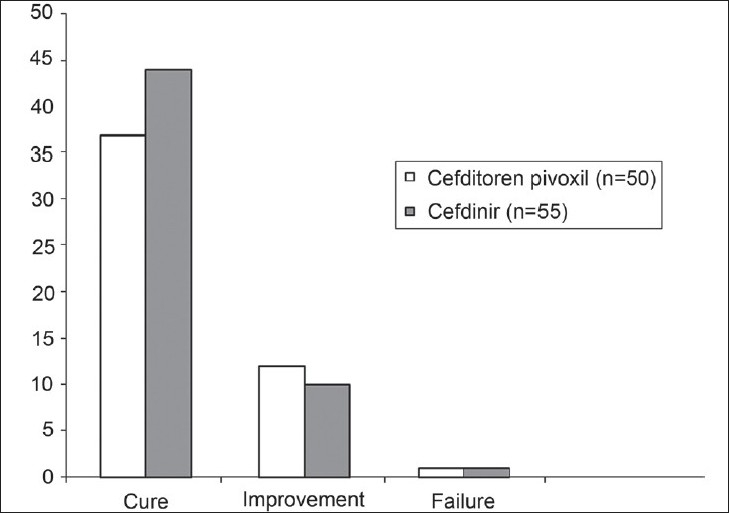
Clinical efficacy of cefditoren pivoxil and cefdinir in the per protocol population (n = 105)

**Figure 3:**
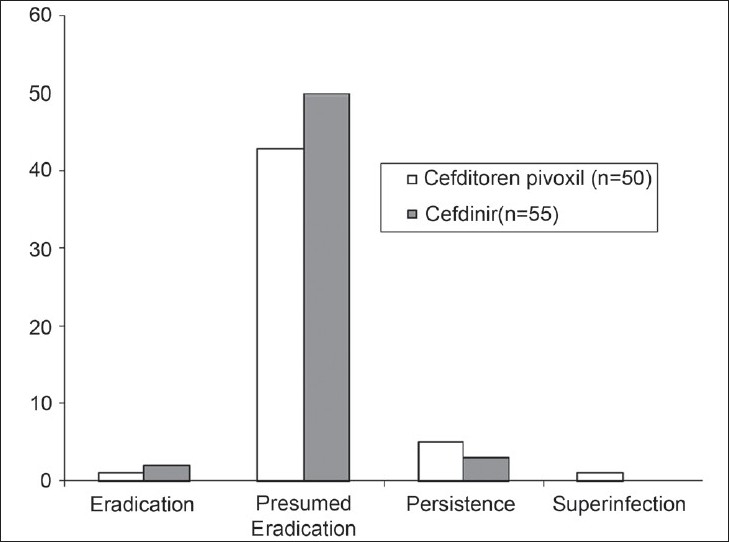
Microbiological efficacy of cefditron pivoxil and cefdinir in the per protocol population (n = 105)

The efficacy data, as measured by clinical and bacteriological outcomes at the end of treatment, are presented in Tables 7–9 and Figures 1–3. In the mITT population, clinical cure or improvement was achieved in 73 of 75 patients (97.33%) treated with cefditoren pivoxil and 76 of 79 patients (96.20%) treated with cefdinir (*P* = 1.0000) [Table 7]. The mean difference and 95% CI for (T – R) was 1.13% (95% CI - 4.44 + 6.70%). In the patients evaluable for PP analysis of clinical outcome, clinical cure or improvement was achieved in 49 of 50 patients (98.00%) treated with cefditoren pivoxil and 54 of 55 patients (98.18%) treated with cefdinir (*P* = 1.0000) [Table 8]. The mean difference and 95% CI for (T – R) was - 0.18% (95% CI - 5.43 + 5.06%). Microbiological eradication or presumed eradication was achieved in 44 of 50 patients (88.00%) treated with cefditoren pivoxil and 52 of 55 patients (94.55%) treated with cefdinir (*P* = 0.3038) [Table 9]. The mean difference and 95% CI for (T – R) was – 8.3% (95% CI - 18.62 + 2.02%). The above differences in both the clinical and microbiological outcome rates between the two drugs were not statistically significant (Fisher's Exact test).

**Table 7:** Summary of clinical efficacy in the mITT population (n = 154)*

Outcome	Cefditoren pivoxil (n = 75) (%)	Cefdinir (n = 79) (%)
Cure	55 (73.33)	59 (74.68)
Improvement	18 (24.00)	17 (21.52)
Failure	2 (2.67)	3 (3.80)

* Data presented as no. of patients with % of patients indicated within the brackets

**Table 8:** Summary of clinical efficacy in the PP population (n = 105)*

Outcome	Cefditoren pivoxil (n = 50) (%)	Cefdinir (n = 55) (%)
Cure	37 (74.00)	44 (80.00)
Improvement	12 (24.00)	10 (18.18)
Failure	1 (2.00)	1 (1.82)

* Data presented as no. of patients with % of patients indicated within the brackets

**Table 9:** Summary of microbiological efficacy in the PP population (n = 105)*

Outcome	Cefditoren pivoxil (n = 50) (%)	Cefdinir (n = 55) (%)
Eradication	1 (2.00)	2 (3.64)
Presumed eradication	43 (86.00)	50 (90.91)
Persistence	5 (10.00)	3 (5.45)
Superinfection	1 (2.00)	0 (0)

* Data presented as no. of patients with % of patients indicated within the brackets

#### Improvement in signs and symptoms

Changes in the clinical signs and symptoms at the end of treatment (Visit 3) are shown in Tables 10 (mITT population) and 11 (PP population). Signs and symptoms at the end of treatment, *i.e*., day 10-12 (Visit 3) showed marked improvement in patients on both cefditoren pivoxil and cefdinir.

**Table 10:** Signs and symptoms at end of study in evaluable patients (mITT) (n = 154)*

Signs and symptoms	Cefditoren pivoxil (75) (%)	Cefdinir (79) (%)
Fever		
Present	5 (6.67)	5 (6.33)
Absent	70 (93.33)	74 (93.67)
Chills		
Present	0 (0)	2 (2.53)
Absent	75 (100)	77 (97.47)
Malaise		
Present	0 (0)	3 (3.80)
Absent	75 (100)	76 (96.20)
Pruritus		
Present	5 (6.67)	7 (8.86)
Absent	70 (93.33)	72 (91.14)
Pain at the site of lesion(s)		
Present	5 (6.67)	6 (7.59)
Absent	70 (93.33)	73 (92.41)
Erythema around the lesion(s)		
Present	5 (6.67)	3 (3.80)
Absent	70 (93.33)	76 (96.20)
Tenderness		
Present	5 (6.67)	5 (6.33)
Absent	70 (93.33)	74 (93.67)
Regional lymphadenopathy		
Present	1 (1.33)	3 (3.80)
Absent	74 (98.67)	76 (96.20)
Ulceration of lesion(s)		
Present	5 (6.67)	4 (5.06)
Absent	70 (93.33)	74 (93.67)
Discharge from lesion(s)		
Present	5 (6.67)	4 (5.06)
Absent	70 (93.33)	75 (94.94)
Crust/Scab formation		
Present	19 (25.33)	24 (30.38)
Absent	56 (74.67)	55 (69.62)
Induration		
Present	4 (5.33)	4 (5.06)
Absent	71 (94.67)	75 (94.94)
Lesions completely healed		
Present	55 (73.33)	59 (74.68)
Absent	20 (26.67)	20 (25.32)
Lesions partially healed		
Present	15 (20.00)	17 (21.52)
Absent	59 (78.67)	62 (78.48)
New lesions		
Present	0 (0)	2 (2.53)
Absent	75 (100)	77 (97.47)

* Data missing for ‘lesions partially healed’ for 1 patient in cefditoren pivoxil group & for ‘ulceration’ for 1 patient in cefdinir group

**Table 11:** Signs and symptoms at end of study in evaluable patients (PP) (n = 105)*

Signs and symptoms	Cefditoren pivoxil (50) (%)	Cefdinir (55) (%)
Fever		
Present	5 (10.00)	4 (7.27)
Absent	45 (90.00)	51 (92.73)
Chills		
Present	0 (0)	1 (1.82)
Absent	50 (100)	54 (98.18)
Malaise		
Present	0 (0)	2 (3.64)
Absent	50 (100)	53 (96.36)
Pruritus		
Present	2 (4.00)	3 (5.45)
Absent	48 (96.00)	52 (94.55)
Pain at the site of lesion(s)		
Present	4 (8.00)	2 (3.64)
Absent	46 (92.00)	53 (96.36)
Erythema around the lesion(s)		
Present	4 (8.00)	2 (3.64)
Absent	46 (92.00)	53 (96.36)
Tenderness		
Present	4 (8.00)	3 (5.45)
Absent	46 (92.00)	52 (94.55)
Regional lymphadenopathy		
Present	0 (0)	1 (1.82)
Absent	50 (100)	54 (98.18)
Ulceration of lesion(s)		
Present	2 (4.00)	2 (3.64)
Absent	48 (96.00)	52 (94.55)
Discharge from lesion(s)		
Present	3 (6.00)	1 (1.82)
Absent	47 (94.00)	54 (98.18)
Crust/Scab formation		
Present	7 (14.00)	14 (25.45)
Absent	43 (86.00)	41 (74.55)
Induration		
Present	3 (6.00)	3 (5.45)
Absent	47 (94.00)	52 (94.55)
Lesions completely healed		
Present	38 (76.00)	43 (78.18)
Absent	12 (24.00)	12 (21.82)
Lesions partially healed		
Present	8 (16.00)	11 (20.00)
Absent	41 (82.00)	44 (80.00)
New lesions		
Present	0 (0)	1 (1.82)
Absent	50 (100)	54 (98.18)

* Data missing for ‘lesions partially healed’ for 1 patient in cefditoren pivoxil group & for ‘ulceration’ for 1 patient in cefdinir group

#### Safety

All the patients recruited into the study were evaluable for safety; six adverse events were reported (two in the cefditoren group and four in the cefdinir group) in three patients (1.69%). Nausea was reported in three patients. Two of these patients also had diarrhea; and one had abdominal pain as well. These AEs were mild to moderate in intensity and resolved without any sequelae. No serious adverse event occurred in this study. Causal relationship was judged as possible by the investigators. No patients were withdrawn from the study due to adverse events.

## Discussion

The present study is the first one performed to assess the clinical efficacy of cefditoren pivoxil in Indian patients with uSSSI. In the present study, 49 of 50 patients (98.00%) treated with cefditoren pivoxil and 54 of 55 patients (98.18%) treated with cefdinir (*P* = 1.0000) achieved clinical cure or clinical improvement (PP population). Bacteriological cure (eradication and presumed eradication) was achieved in 88.00% patients treated with cefditoren pivoxil and 94.55% patients treated with cefdinir. In the mITT population 97.33% patients treated with cefditoren pivoxil and 96.20% patients treated with cefdinir achieved clinical cure or clinical improvement. The differences in clinical and bacteriological outcomes between cefditoren pivoxil and cefdinir were not statistically significant (Fisher's Exact test). The most commonly observed adverse events (AEs) in this study were nausea and diarrhea, which resolved without therapy discontinuation or any sequelae. The results of the present study are similar to the results reported in published clinical studies where cefditoren pivoxil was compared with cefuroxime axetil and cefadroxil.[1,3]

## Conclusion

Cefditoren pivoxil 200 mg *b.i.d*. is effective and well tolerated in the treatment of uSSSI in Indian patients. Clinical and bacteriological efficacy of cefditoren pivoxil in the treatment of uSSSI was comparable to that of cefdinir.
